# Modelling severe COVID-19 in TLR3-mutated hiPSCs-derived lung organoids

**DOI:** 10.1038/s41420-025-02936-5

**Published:** 2025-12-26

**Authors:** Andrea Latini, Paola Spitalieri, Federica Centofanti, Barbara Rizzacasa, Donatella Amatore, Giorgia Grilli, Riccardo De Santis, Lorenzo Vaccaro, Vito Luigi Colona, Giulio Puleri, Anna Maria Nardone, Michela Biancolella, Elena Campione, Loredana Sarmati, Paola Rogliani, Davide Cacchiarelli, Antonio Novelli, Federica Sangiuolo, Florigio Lista, Giuseppe Novelli

**Affiliations:** 1 Department of Biomedicine and Prevention, Genetics Section, University of Rome Tor Vergata, Rome, Italy; 2 UniCamillus, Saint Camillus International University of Health Sciences, Rome, Italy; 3 Defence Institute for Biomedical Sciences, Rome, Italy; 4 Department of Public Health and Infectious Diseases, Sapienza University of Rome, Rome, Italy; 5 Telethon Institute of Genetics and Medicine (TIGEM), Armenise/Harvard Laboratory of Integrative Genomics, Pozzuoli, Italy; 6 Department of Translational Medicine, University of Naples “Federico II”, Naples, Italy; 7 Unit of Neurorehabilitation, Bambino Gesù Children’s Hospital, IRCCS, Rome, Italy; 8 Department of Chemical-Toxicological and Pharmacological Evaluations of Drugs, Catholic University Our Lady of Good Counsel, Tirane, Albania; 9 Medical Genetics Laboratory, Policlinico Tor Vergata Hospital, Rome, Italy; 10 Department of Biology, University of Rome “Tor Vergata”, Rome, Italy; 11 Dermatology Unit, Department of Systems Medicine, University of Rome Tor Vergata, Rome, Italy; 12 Clinical Infectious Diseases, Department of System Medicine, Tor Vergata University, Rome, Italy; 13 Unit of Respiratory Medicine, Department of Experimental Medicine, University of Rome Tor Vergata, Rome, Italy; 14 Genomics and Experimental Medicine Program, Scuola Superiore Meridionale (SSM, School of Advanced Studies), Naples, Italy; 15 Laboratory of Medical Genetics, Translational Cytogenomics Research Unit, Bambino Gesù Children’s Hospital, IRCCS, Rome, Italy; 16 Giovanni Lorenzini Medical Foundation, Milan, Italy

**Keywords:** Genetics research, Genotype

## Abstract

Clinical variability in COVID-19 is partly explained by host genetic factors, including inborn errors of immunity. We investigated a patient with a heterozygous nonsense mutation in the *TLR3* gene (p.Trp769*) by generating human-induced pluripotent stem cells (hiPSCs) and differentiating them into lung organoids (hLORGs). TLR3-mutated hLORGs showed reduced basal expression of *TLR3* and downstream signaling genes. Following infection with a pseudotyped SARS-CoV-2 virus and live SARS-CoV-2, RNA-Seq and qPCR analyses revealed significant upregulation of fibrinogen genes (*FGA*, *FGG*), which are associated with severe COVID-19. Interestingly, *TLR3* expression remained inducible upon infection, despite the loss-of-function mutation. Our patient-derived hLORG model recapitulates the pathophysiological features of the patient and provides a platform to investigate host–virus interactions and test targeted therapies for genetically at-risk individuals.

## Introduction

Since the onset of the COVID-19 pandemic in December 2019, more than 300 million people have been infected with SARS-CoV-2, causing more than 6 million deaths worldwide [1]. The consequences of exposure to SARS-CoV-2 vary greatly across individuals, with outcomes ranging from asymptomatic infection to life-threatening disease, with a broad spectrum of clinical presentations in between. This wide inter-individual clinical variability represents a scientific and clinical challenge [2]. The major epidemiological risk factor for life-threatening COVID-19 pneumonia is age, with a risk that doubles every 5 years. However, a substantial inter-individual clinical variability persists within demographic category. Recent papers have highlighted that approximately 3% of patients with critical COVID-19 pneumonia have inborn errors of immunity (IEI) that impair the TLR3-, TLR7-, and IRF7-dependent type I interferon (IFN) immunity, particularly in individuals under 65 years of age [3]. Additionally, at least another 10% of patients carry pre-existing autoantibodies (auto-Abs) neutralizing type I INFs, especially among patients over 65 years of age [3, 4]. Furthermore, X-linked TLR7 deficiency has been identified as the cause of at least 1% of critical cases in men under 65 years of age [5]. These findings further suggest that the pathogenesis of life-threatening COVID-19 pneumonia in most patients occurs in two stages, with an initial insufficient type I IFN response that allows the virus to spread, subsequently triggering excessive inflammation [6]. Indeed, insufficient type I IFN levels permit the virus to replicate early in the upper respiratory tract and spread to the lungs and bloodstream, triggering a strong leukocyte response aimed at resolving the widespread infection but at the cost of severe inflammation [7].

The development of cellular models that mimic human tissues has revolutionized biomedical research, providing a crucial platform for studying disease mechanisms and testing therapeutic interventions. Among these models, organoids have emerged as a powerful tool, offering a three-dimensional culture system that recapitulates key structural and functional aspects of human organs [8]. These models can also be a valuable support for investigating diseases associated with genetic mutations, as they allow for the pathophysiological study of the specific organ in which the mutated gene is most highly expressed. One of the key areas where organoid technology has been widely applied is in the study of respiratory diseases, including COVID-19. Lung organoids offer an in vitro model that replicates human lung tissue, enabling researchers to investigate host-pathogen interactions.

A study by Zhang et al. highlighted the significance of genetic variations in the interferon pathway in influencing the host response to SARS-CoV-2 infection [3]. This study identified several mutations associated with impaired antiviral responses, underscoring the role of innate immunity in susceptibility to COVID-19. Further investigation of the functional consequences of these variants using lung organoid models could provide critical insights into their impact on antiviral defense mechanisms and potential therapeutic targets. Among subjects carrying pathogenic variants in the interferon pathway and affected by a severe form of COVID-19, we identified a male subject carrying the nonsense p.(Trp769*) variant in the *TLR3* gene, a key component of viral DNA recognition and innate immune response [3]. Given the crucial role of TLR3 in recognizing viral double-stranded (dsRNA) and triggering IFN responses, this mutation may contribute to impaired immune responses and increased disease severity. Therefore, our study aims to gain knowledge about human genetics and immune responses underlying the different clinical manifestations of SARS-CoV-2 infection, facilitating translation into clinical practice. Using patient-derived lung organoids, we aim to determine whether this experimental model can serve as a reliable model to reproduce the pathophysiological features associated with the rare *TLR3* p.(Trp769*) variant, providing a platform to investigate the mutation-specific effects on antiviral immune responses.

## Results

### Clinical features at time of SARS-CoV-2 infection

At the time of initial clinical evaluation, the patient was a 77-year-old male admitted to the Pneumonology-Cardiology units of the University Hospital Policlinico Tor Vergata of Rome for a 22-day hospitalization without the need for intensive care. His medical history includes chronic anemia, congestive heart failure, first-degree atrioventricular block, chronic obstructive pulmonary disease (treated with home-based therapy including diuretics and corticosteroids), and a history of smoking. During hospitalization, he received macrolide antibiotics, antiviral therapy, therapeutic-dose heparin, and non-invasive oxygen therapy. At admission (April 1, 2020), laboratory findings revealed leukocytosis (7.82 × 10³/mL) with marked neutrophilia (79.7%) and lymphopenia (13.9%). C-reactive protein (CRP) was significantly elevated (140.8 mg/L; reference range: 0–5), as were lactate dehydrogenase (LDH, 469 U/L; reference range: 125–220), fibrinogen (701 mg/dL; reference range: 200–400), and D-dimer (1501 ng/mL; reference range: 0–500). Hypoalbuminemia was present (2.52 g/dL; reference range: 3–5.5), while serum creatinine levels remained within normal limits. Inflammatory cytokine profiling revealed elevated IL-6 levels (32.6 pg/mL; normal range <12.4), with TNF-α levels within the normal range (26.4 pg/mL; normal range <50). Chest imaging demonstrated interstitial ground-glass opacities consistent with viral pneumonia. Electrocardiogram (ECG) showed sinus rhythm at 80 beats per minute (bpm), first-degree AV block (PR interval: 240 ms), a QRS duration of 120 ms, QT interval of 400 ms (QTc: 468 ms), and flat T waves in the inferolateral leads, with a non-dynamic ST segment. The patient was classified as NYHA Class II, indicating mild limitation of physical activity, with symptoms such as fatigue, palpitations, or dyspnea during ordinary activity, but comfortable at rest. The patient was also sequenced to find a rare genome variant in inborn errors of Toll-like receptor 3 (TLR3)– and interferon regulatory factor 7 (IRF7)–dependent type I interferon (IFN) immunity, which underlie life-threatening influenza pneumonia. As reported in Zhang et al. (2020), we identified in this patient the nonsense NM_003265.3:c.2307 G > A; p.(Trp769*) variant in the *TLR3* gene [3].

### Development and characterization of TLR3 patient-specific human-induced pluripotent stem cell (hiPSCs)

**Fig. 1:**
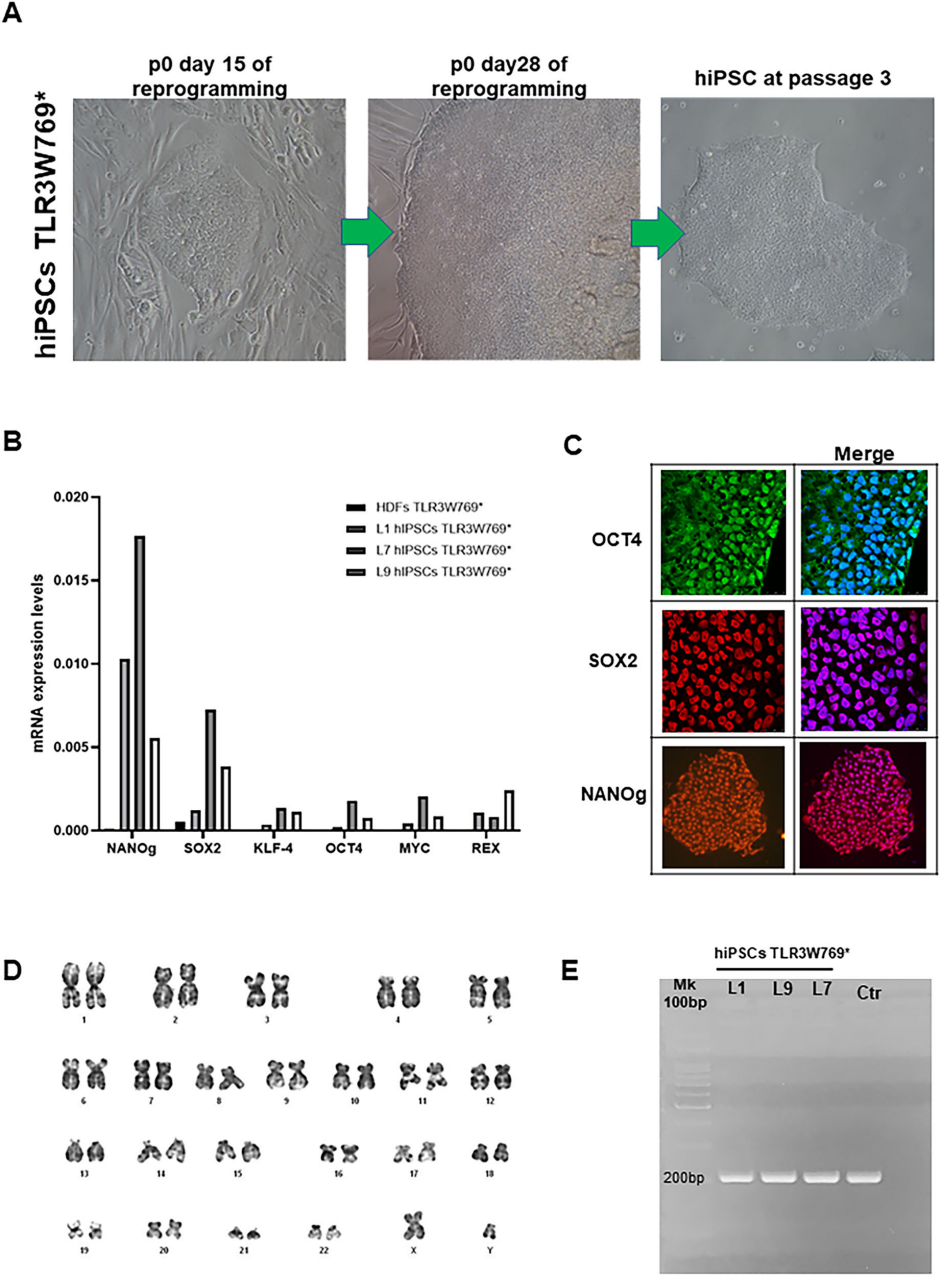
Generation and characterization of TLR3 patient-specific human-induced pluripotent stem cells (hiPSCs TRL3W769*). **A** Main point of protocol used for hiPSCs generation: day 15 of reprogramming during which mesenchymal-epithelial transition occurs; day 28, during which hiPSC cell colonies were formed and grew enough to be isolated and propagated. Representative hiPSCs line morphology at passage 3 (p3). **B**, **C** Three hiPSCs lines (L1, L7 and L9) have been characterized at p3 for the expression of pluripotency markers *OCT4, SOX2, NANOG, KLF4, REX1* and *c-MYC* by RT-PCR and immunofluorescence analyses**. D** Representative hiPSCs line showed normal karyotype by G-banding analysis**. E** Mycoplasma detection by PCR. The only band was detected at 200 bp, which represents the internal control of the reaction, which refers to no mycoplasma contamination. Ctr: is a control reaction without a template.

Human dermal fibroblasts (HDFs) from the *TLR3* mutated patient were reprogrammed using a non-integrating, self-replicating RNA reprogramming vector encoding all four reprogramming factors (Oct-3/4, Klf-4, Sox2, Glis1, c-Myc). Thirteen hiPSCs lines were generated, of which three were selected for further analysis (Table 1). All hiPSCs exhibited an undifferentiated morphology with compact, refractile colonies, defined borders, and a high proliferation rate (Fig. 1A). Successful reprogramming was further confirmed by RT-qPCR analysis using primers specifically designed to detect endogenous reprogramming transcription factors (*OCT4, NANOG, SOX2, KLF4, c-MYC* and *REX-1*) (Fig. 1B, C). hiPSCs-derived lines showed clear expression of pluripotency markers compared to fibroblasts from the same patient, confirming their stemness. Moreover, karyotype analysis confirmed a normal male 46, XY diploid profile (Fig. 1D). Molecular analysis of DNA polymorphisms (STR) across 24 *loci* confirmed that all three hiPSCs-derived lines originated from the same donor (HDFs TLR3W769*) (Table S1). No mycoplasma contamination was detected in any of the hiPSCs-derived lines (Fig. 1E).

**Table 1:** hiPSCs TLR3W769* Cell line features.

Unique stem cell line identifier	TLR3W769*
Type of cell line	hiPSC
Origin	Human
Additional origin info	Age: 80 Sex: Male Ethnicity: Caucasian
Cell Source	Dermal Fibroblast
Method of reprogramming	Kit for generating hiPS cells using ReproRNA™-OKSGM, a non-integrating, self-replicating RNA reprogramming vector (StemCell Technologies)
Clonality	Clonal
Cell culture system used	Cells cultured on hESC Matrigel®-coated plates in mTeSR Plus Medium (StemCell Tecnologies)
Gene/Locus	*TLR3*; 4q35.1; NM_003265.3:c.2307 G > A; p.(Trp769*)

**Fig. 2:**
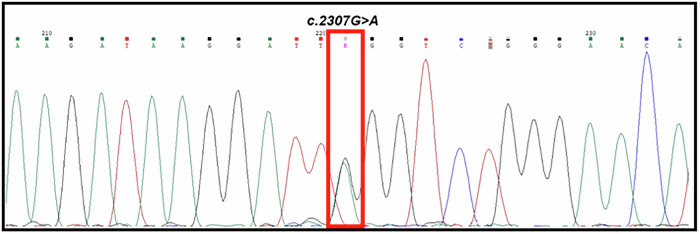
Sanger sequencing of hiPSCs TLR3W769*. The electropherogram confirms the presence of the heterozygous variant NM_003265.3:c.2307 G > A; p.(Trp769*) in the *TLR3* gene.

We also genotyped the hiPSCs-derived lines to confirm the presence of the specific genomic alteration in the *TLR3* gene, and verified that they carried the same mutation as the donor patient: NM_003265.3:c.2307 G > A; p.(Trp769*) (Fig. 2).

Finally, telomere analysis revealed a relative increase in telomere length in all three hiPSC-derived lines compared to fibroblasts from the 80-year-old patient. Values were normalized to telomere expression levels from a 46-year-old reference individual (Fig. S1).

### Derivation and differentiation into human 3D lung organoids (hLORGs)

**Fig. 3:**
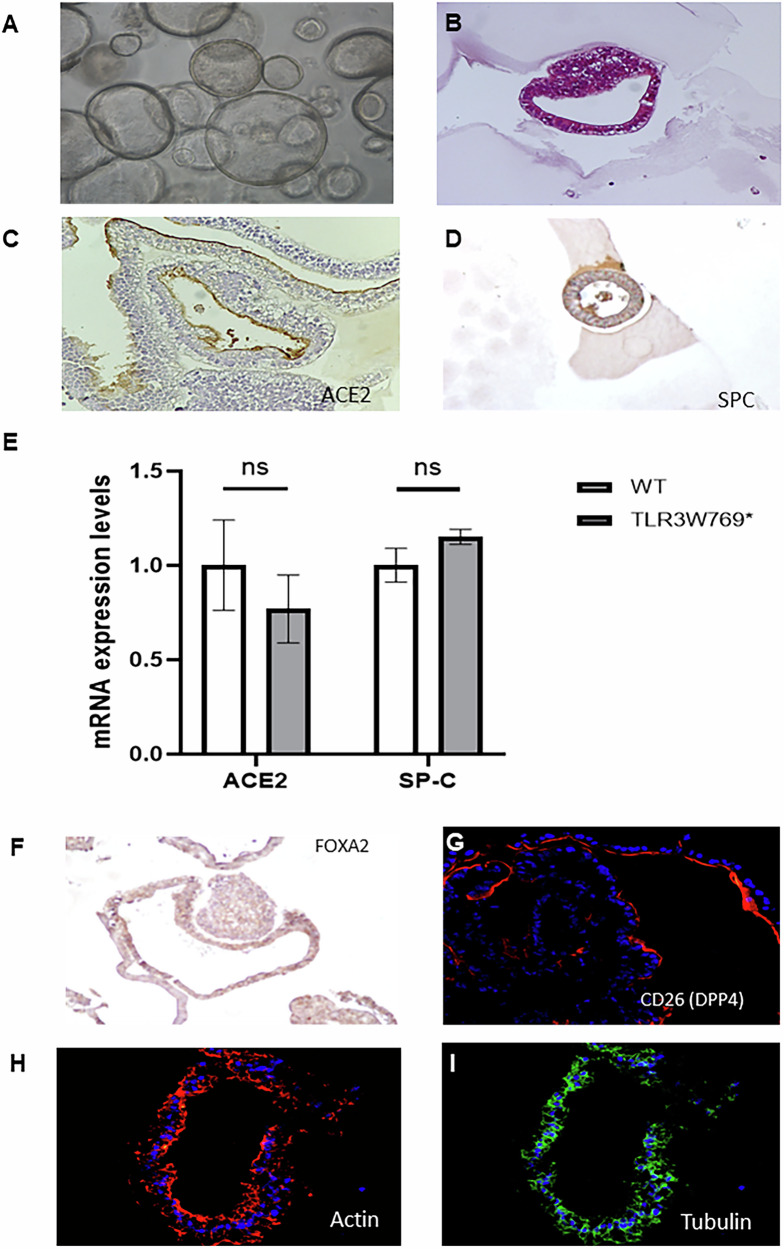
Characterization of human lung organoids (hLORGs) derived by hiPSCs TLR3W769*. **A** Phase–contrast microscopy of alveolospheres embedded in 3D Matrigel at day 51 of culture. Scale bar 200 µm. **B** Haematoxylin and eosin-stained hLORG cross-section showing the typical epithelial morphology. Scale bar 50 µm. **C–E** ACE2, SFTPC protein and gene expression in hLORGs at day 51 of differentiation by immunohistochemistry and qPCR analysis. **F** FOXA2 protein expression in hLORGs at day 51 of differentiation by immunohistochemistry. Scale bar 50 µm. **G** DPP4 protein expression in hLORGs at day 51 of differentiation by immunofluorescence. Scale bar 50 µm. **H**, **I** Immunofluorescence images show the overall actin (red) and α-tubulin distribution (green) of an hLORG displaying a prominent epithelial structure (nuclei, blue). Scale bar 50 µm and 25 µm, respectively.

Once the hiPS lines were fully characterized, we differentiated them into lung organoids to investigate the effect of the *TLR3* mutation. We established self-renewing epithelial sphere cultures in 3D Matrigel, comprising organ-specific cell types that self-organize through spatially regulated lineage commitment. We successfully obtained 3D hLORG models, including AT2-like cells that express ACE2 and DPP4, along with surfactant protein SPC and the transcription factor FOXA2. The latter plays a crucial role in lung morphogenesis and cell differentiation during lung development, particularly in the regulation of surfactant production, which is essential for proper lung function [9] (Fig. 3).

### TLR3 signaling pathway expression in fibroblasts and hLORGs

**Fig. 4:**
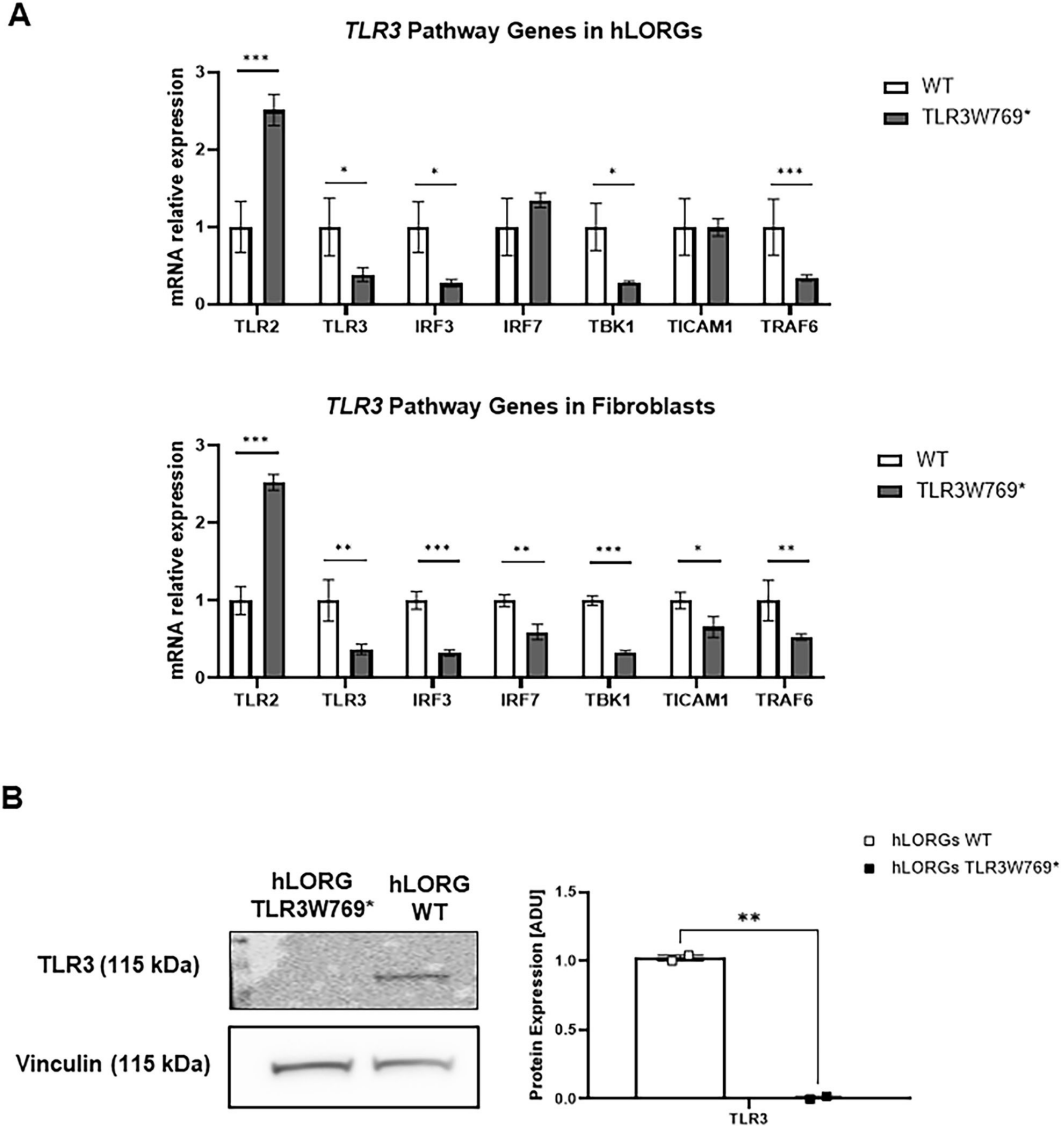
Expression of the TLR3 pathway in wild-type and TLR3W769* patients. Relative mRNA expression of *TLR3* pathway-related genes in **A** wild type and TLR3W769* hLORGs (Upper panel) and wild type and TLR3W769* HDFs (Lower panel). The bar graph shows the expression of genes relating to the *TLR3* Pathway quantified by qRT-PCR. The results are represented as the mean ± SEM (* *p* < 0.05; *** *p* < 0.001; **** *p* < 0.0001; by one-way ANOVA test, *n* = 3). **B** Blot and densitometric analysis of TLR3 in wild type and TLR3W769* hLORGs. The average was evaluated in at least two independent experiments and is reported as means ± SEM; (** *p* < 0.01 estimated by a one-way ANOVA test, *n* = 2). Abbreviations: ADU arbitrary densitometric units.

To assess the potential impact of the NM_003265.3:c.2307 G > A; p.(Trp769*) mutation in *TLR3* gene, we used qPCR to compare the expression levels of key genes related to the *TLR3* pathway in both TLR3W769* and WT HDFs and hLORGs. In the patient-derived tissues, we observed a reduced expression of the *TLR3* gene expression. Furthermore, the patient also showed lower expression levels of *IRF3*, *TBK1*, and *TRAF6* genes in both HDFs and hLORGs compared to the control. Interestingly, expression levels of another Toll-like receptor, *TLR2*, were increased in the patient relative to the control, suggesting that the mutation specifically affects the TLR3 pathway and may trigger a compensatory mechanism (Fig. 4A). Moreover, Western blot analysis was performed to evaluate TLR3 protein abundance in WT and TLR3W769* hLORGs. This analysis revealed that TLR3 protein levels were undetectable in TLR3W769* hLORG compared to WT hLORGs (**p < 0.01; Fig. 4B), indicating that TLR3 protein was not expressed in lung organoids despite the heterozygous state of the variant.

### RNA-Seq analysis in WT and TLR3W769* hLORGs infected with VSV-pseudo SARS-CoV-2 virus

To study the alteration of TLR3 signaling in hLORGs carrying the *TLR3* mutation p.(Trp769*) compared to WT hLORGs, the organoids were infected with the VSV-pseudotyped SARS-CoV-2 Omicron XBB.1.5 and cells were harvested for analysis. Transduction efficiency was quantified by measuring virus-encoded luciferase activity in hLORGs infected for 48 h (Fig. S2). Both hLORGs showed VSV-pseudotyped SARS-CoV-2 Omicron XBB.1.5 infection with high transduction efficiency.

**Fig. 5:**
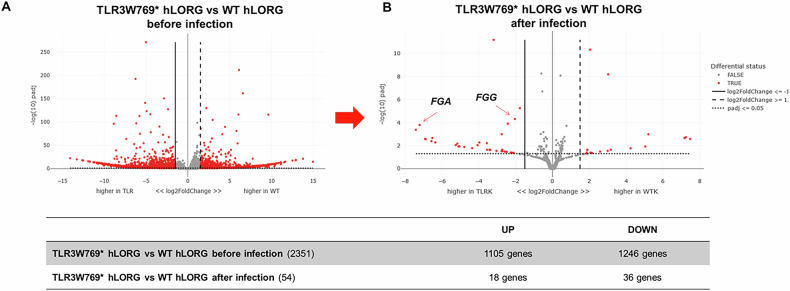
Volcano plot of RNA-Seq analysis in wild type and TLR3W769* hLORGs infected with VSV-pseudo SARS-CoV-2 virus. **A** The Volcano plot illustrates the significant mRNAs that we identified as down-regulated in wild type and TLR3W769* hLORGs before infection, positioned on the left (down-regulated) and above the vertical line (statistically significant), based on significant -log(10)padj and log2fold-change. **B** The Volcano plot depicts the significant mRNAs that we discovered to be down-regulated in wild type and TLR3W769* hLORGs following infection, placed on the left (down-regulated) and above the vertical line (statistically significant), according to significant −log(10)padj and log2fold-change. The vertical lines indicate no change in gene expression (log2fold-change |1 | = 0). The red dotted lines represent an established threshold or boundary for fold change, with a default setting of 2. The solid vertical line signifies a selected threshold for the −log(10)padj and statistical significance, with the default setting set at 0.05. Statistical significance versus fold change is illustrated on the y- and x-axes, respectively.

We subsequently performed RNA-Seq analysis on organoids both before and after infection with the VSV-pseudotyped SARS-CoV-2. This analysis revealed that WT and TLR3W769* hLORGs exhibited distinct gene expression profiles prior to infection (Fig. 5A), with KEGG pathway enrichment highlighting a significant overrepresentation of differentially expressed genes involved in the Coronavirus disease pathway (hsa05171; FDR = 1.8029e-12 and *P* = 1.0273e-14). After infection, the expression profiles were largely similar, except for 54 differentially expressed genes (Table S2). In particular, among these, the analysis revealed higher expression of the Fibrinogen alpha chain (*FGA*) and Fibrinogen gamma chain (*FGG*) genes in TLR3W769* hLORGs following VSV-pseudotyped SARS-CoV-2 Omicron XBB.1.5 infection, both belonging to the fibrinogen pathway (Fig. 5B). These data suggest a potential link between the *TLR3* mutation and altered fibrinogen production in response to viral infection.

### Validation of expression data in hLORGs infected with VSV Pseudo and live SARS-CoV-2

**Fig. 6:**
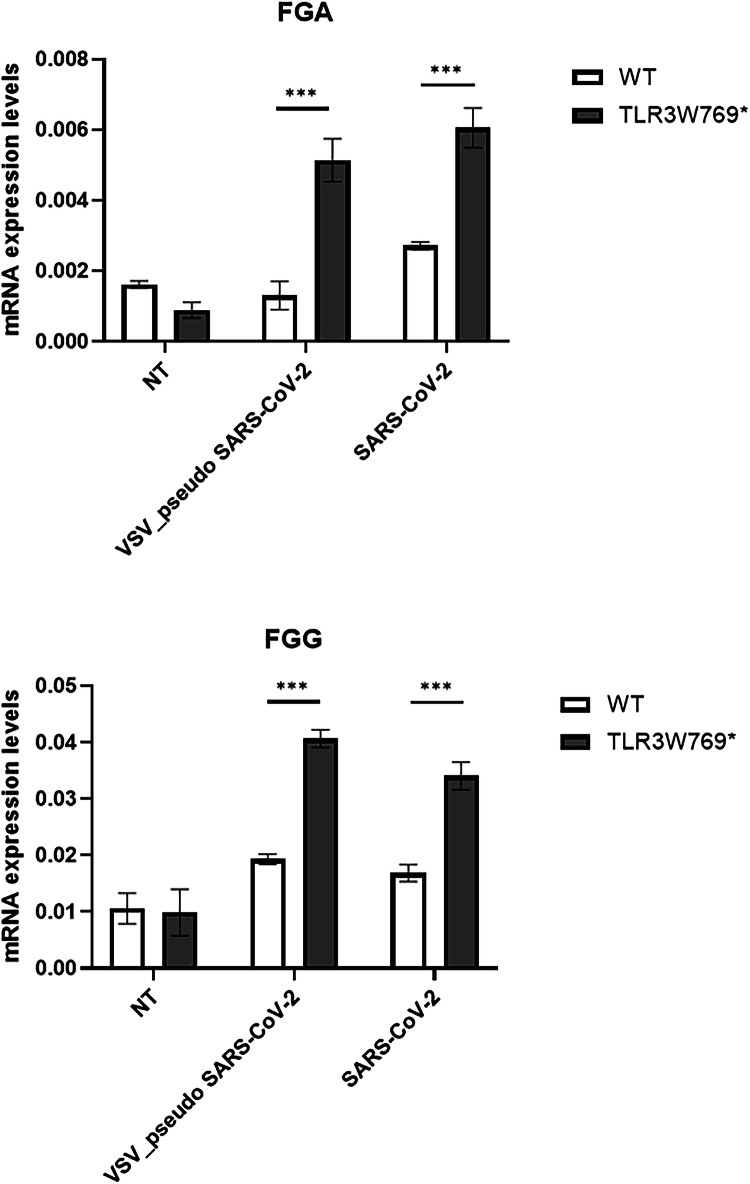
Expression of Fibrinogen genes in wild type and TLR3W769* hLORGs. Relative mRNA expression of *FGA* (Upper panel) and *FGG* (Lower panel) genes in wild type and TLR3W769* hLORGs after 48 hpi with both VSV-pseudo and SARS-CoV-2 viruses and without (NT) virus. The bar graph shows the expression of fibrinogen genes quantified by qRT-PCR. The results are represented as the mean ± SEM (*** *p* < 0.00; by one-way ANOVA test, *n* = 3). Abbreviations: hpi hours after infection.

To confirm the RNA-Seq data, we validated by qPCR the expression of two fibrinogen genes and we confirmed a consistent increase in the levels of the *FGA* and *FGG* genes in TLR3W769* hLORGs at 48 h post-infection with VSV-pseudotyped SARS-CoV-2. To further validate this upregulation, both TLR3W769* and WT hLORGs were infected with live SARS-CoV-2, confirming the previously reported results (Fig. 6). According to the literature, high expression of these two genes is associated with severe COVID-19 [10]. Further studies are underway to elucidate the link between the *TLR3* mutation and the regulation of these genes.

**Fig. 7:**
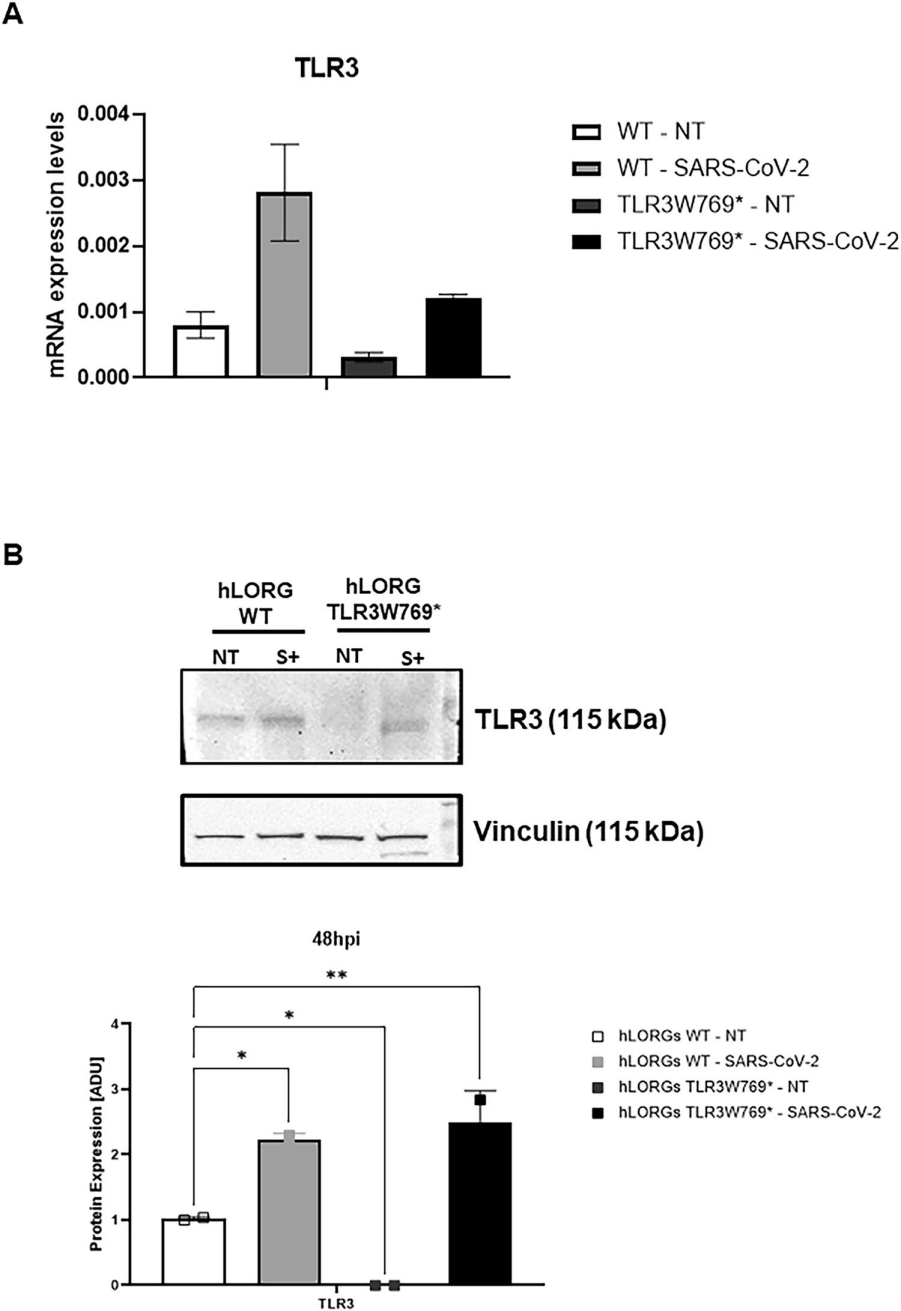
Expression of TLR3 in wild type and TLR3W769* hLORGs. **A** Relative mRNA expression of *TLR3* genes in wild type and TLR3W769* hLORGs after 48 hpi with SARS-CoV-2 and without (NT) virus. The bar graph shows the expression of *TLR3* genes quantified by qRT-PCR. The results are represented as the mean ± SEM (*p < 0.05; ***p < 0.001; ****p < 0.0001; by one-way ANOVA test, *n* = 3). **B** Blot and densitometric analysis of TLR3 protein in wild type and TLR3W769* hLORGs hLORGs after 48 hpi with SARS-CoV-2 and without (NT) virus. The average was evaluated in at least two independent experiments and is reported as means ± SEM; (* *p* < 0.05 and ** *p* < 0.01 estimated by a one-way ANOVA test, *n* = 2). Abbreviations: hpi hours post-infection, ADU arbitrary densitometric units.

Although we observed a significant difference in basal *TLR3* levels between WT and TLR3W769* hLORGs (Fig. 4A), RNA-Seq profiles did not reveal any differential *TLR3* expression between the two models after VSV-pseudotyped SARS-CoV-2 infection. Therefore, we investigated TLR3 expression at both gene and protein levels in hLORGs before and after SARS-CoV-2 infection. We observed an increase in *TLR3* gene expression in both control and patients’ samples after 48 hours of infection (Fig. 7A). Similarly, although TLR3 protein was undetectable prior to infection in the mutated patient (Figs. 4B, 7B), TLR3 protein levels increased 48 h post-infection in both control and patient (Fig. 7B), confirming the lack of difference observed in RNA-Seq analyses after VSV-pseudotyped SARS-CoV-2 infection.

## Discussion

The primary aim of our study is to demonstrate that lung organoids derived from a patient carrying a rare variant, specifically the loss-of-function p.(Trp769*) mutation in *TLR3* gene, can serve as a reliable platform for investigating patient-specific pathophysiological mechanisms. SARS-CoV-2 and the ensuing COVID-19 pandemic have revealed high heterogeneity in clinical outcomes ranging from asymptomatic infection to life-threatening pneumonia and acute respiratory distress syndrome [4, 11]. Severe cases are often characterized by a dysregulated immune response, typically involving impaired early induction of type I IFN followed by excessive inflammation [12]. Rare inborn errors of immunity have been increasingly recognized as contributing factors to this variability, with *TLR3* genetic variants among those associated with life-threatening forms of COVID-19 [13, 14]. TLR3 is a key endosomal receptor responsible for recognizing dsRNA, a molecular intermediate formed during the replication of numerous viruses, including coronaviruses [15]. Although SARS-CoV-2 is a positive-sense single-stranded RNA virus, its replication cycle involves the formation of dsRNA intermediates, and TLR3 plays a critical role as a sensor in the host’s defense against this virus [12]. Its activation typically induces the production of type I IFNs, establishing an antiviral state and promoting the generation of pro-inflammatory cytokines that facilitate pathogen clearance. The balance between antiviral activity and pro-inflammatory responses mediated by TLR3 is likely a crucial determinant of disease outcome. Our findings suggest that the TLR3 variant p.(Trp769*) impairs the basal activity of this receptor, thus delaying the initiation of antiviral defense.

It is important to note that no morphological differences were observed between WT and patient-derived hLORGs, neither under basal conditions nor following infection. On the contrary, at the transcriptional level, our results showed that the mutation leads to reduced basal *TLR3* gene expression with a consequent downregulation of key genes in the associated signaling cascade. This impairment may predispose to a delayed innate immune response, thereby contributing to dysregulated inflammation. Although patient-derived hLORGs exhibit significantly lower basal *TLR3* expression compared to WT controls, they appear to retain the ability to upregulate TLR3 to levels comparable to those observed in WT organoids following viral infection. This supports the hypothesis of an adaptive cellular response to viral infection, or a virus-driven modulation of the host gene expression machinery [15–21]. These data suggest that the mutation does not impair the gene’s inducibility but may alter its basal signaling efficacy, potentially delaying the initial antiviral response. Such early impairment could be critical, as prompt pathogen recognition is essential for effective viral containment. This interpretation is supported by previous studies underscoring the role of TLR3 in antiviral defense. For instance, Zhang et al. reported rare *TLR3* loss-of-function (LoF) variants in individuals with life-threatening COVID-19, showing that these mutations disrupted type I IFN production in response to SARS-CoV-2 [3]. Similarly, Bortolotti et al. found that *TLR3* knockout mice exhibited exacerbated disease severity and abnormal cytokine profiles after SARS-CoV-2 infection [16]. Furthermore, in vitro studies in chicken fibroblast cell line DF-1 showed that targeted knockout of *TLR3* impaired induction of type I IFN and downstream antiviral genes upon stimulation with RNA ligands such as poly(I:C) [22]. These findings confirm that TLR3 is essential for initiating a robust innate immune response to viral RNA, and its absence can significantly impair cellular antiviral signaling.

Importantly, our organoid model reproduced transcriptional signatures consistent with clinical manifestations of severe COVID-19, which occurred in our TLR3 patient. In particular, we observed upregulation of *FGA* and *FGG* genes in the *TLR3*-mutated hLORGs upon viral challenge, suggesting that the impaired early antiviral response due to TLR3 deficiency could lead to a more pronounced and potentially dysregulated inflammatory response. From a clinical perspective, this upregulation is of particular interest. Fibrinogen is a key player in the coagulation cascade and an acute-phase reactant often elevated in systemic inflammation. Increased expression of these genes may contribute to the prothrombotic state and exaggerated pulmonary inflammation commonly observed in severe COVID-19 cases [23–27]. This supports the notion that dysfunctional innate immune sensing, such as that caused by TLR3 defect, might not only weaken antiviral defense but also predispose to immune-thrombotic complications commonly seen in patients.

The capacity to generate lung organoids from hiPSCs, particularly those derived from individuals with specific genetic backgrounds or engineered to incorporate particular mutations, offers a substantial advantage for exploring the role of human genes such as *TLR3* in the context of SARS-CoV-2 infection. Although hiPSC-derived organoids recapitulate key molecular and cellular features of human lung tissue, they represent a simplified model of the organ in vivo. Although previous studies have demonstrated high degree of transcriptomic and phenotypic similarity between lung organoids and patient lung epithelium, differences in cellular composition and microenvironmental signaling remain [8, 28]. Nevertheless, considering the potential involvement of TLR3 in the immune response to SARS-CoV-2 and the documented association of *TLR3* genetic variants with COVID-19 severity [29], the use of *TLR3*-mutated hiPSC-derived lung organoids establishes a compelling in vitro system to investigate pathophysiological mechanisms underlying severe disease outcomes.

Finally, the methodological approach established here holds promise beyond the *TLR3* p.(Trp769*) variant. Patient-derived organoids could be employed to investigate additional rare variants involved in innate immunity, allowing a systematic exploration of their role in viral susceptibility. Moreover, these models provide a preclinical platform for testing therapeutic strategies aimed at restoring impaired immune responses, such as IFN-based therapies, or mitigating downstream pathological effects, including hyperinflammation and pro-thrombotic responses.

It is important to highlight that the present study is based on organoids derived from a single individual carrying a very rare variant (ƒ = 0.000002478, gnomAD v4.1.0) of the TLR3 gene. Although this personalized model allows for detailed exploration of patient-specific mechanisms, further validation in other subjects will be necessary to confirm the generalizability of our findings.

In conclusion, our study validates patient-derived lung organoids as a faithful model of the TLR3 p.(Trp769*)-associated pathophysiology, demonstrating how rare genetic variants can be functionally reproduced ex vivo. These findings support the use of personalized organoid models to explore gene-disease associations and potential targeted interventions (*e.g*., fibrinogen-targeted therapies or IFN supplementation) in COVID-19 and related viral diseases. Beyond COVID-19, such models could be instrumental for investigating other mutations in innate immune pathways and for testing targeted therapeutic interventions in a patient-specific context.

## Material and methods

### hiPS cells reprogramming from human dermal fibroblasts (HDFs)

The *TLR3* mutant patient (TLR3W769*) underwent a dermal biopsy in 2023 at the age of 80 (Table 1). Before participation, informed written consent was obtained. The project was approved by the Committees on Health Research Ethics of the University Hospital Policlinico Tor Vergata of Rome (protocol n. 2932/2017) and was conducted in accordance with the Declaration of Helsinki.

Skin biopsies were processed as previously reported, and human dermal fibroblasts were obtained [30]. The outgrowing fibroblasts were expanded in conventional serum-containing culture medium up to passage 4. To generate hiPS cells, we used the ReproRNA™-OKSGM kit (StemCell Technologies), a non-integrating, self-replicating RNA reprogramming vector encoding all four reprogramming factors (Oct-3/4, Klf-4, Sox2, Glis1, and c-Myc) along with a puromycin-resistance cassette in a single construct.

Approximately 1 × 10^5^ HDFs have been seeded on 6-well culture plates with Corning® Matrigel® hESC-Qualified Matrix in Fibroblast Growth Medium. The cells were transfected using the ReproRNA-OKSGM vector (STEMCELL Technologies, #05930) according to the manufacturer’s protocol and grown from day 2 to day 8 with Growth Medium with Puromycin. Valproic acid [0.5 mM], a histone deacetylase (HDAC) inhibitor, was added to enhance reprogramming efficiency. From days 8 to 14, the medium was changed to ReproTeSR™ with B18R and then without B18R until the hiPSC colony formed. hiPSC colonies were manually transferred to pre-treated Corning® Matrigel® hESC-Qualified Matrix Matrigel-coated 30 mm dish for isolation and expansion and maintained in mTeSR Plus Medium with Y-27632 ROCK inhibitor (STEMCELL Technologies) at 37 °C and 5% CO_2_, maintaining the stability over 20 or more passages.

Three cell lines (L1, L7, and L9) at passage 3 (p3) were used to characterize iPSCs through molecular and immunofluorescence analyses.

### RT-qPCR

The total RNA was extracted from cells using Trizol Reagent (Invitrogen Life Technologies Corporation, Carlsbad, CA, USA), following manufacturer’s instructions. One μg of RNA was reverse transcribed with the High-Capacity cDNA Reverse Transcription Kit (Life Technologies Corporation, Foster City, CA, USA) and expression analysis was performed by RT-qPCR (SYBR Green Assay, Applied Biosystems, Foster City, CA, USA). GAPDH was used as a reference gene. Primer sequences are available upon request. The 2^-(ΔCt)^ and comparative ΔΔCt methods were used to quantify relative gene expression levels.

### DNA extraction and Sanger sequencing

Genomic DNA was extracted from cells through a manual lysis phase, followed by automated extraction using the “Maxwell® 16 MDx Instrument” (Promega, WI, USA) following the kit “DNA IQ™ Casework Pro Kit for Maxwell® 16” (Promega, WI, USA) as instructed by the manufacturer. DNA concentration and quality were assessed with the NanoDrop ND-1000 Spectrophotometer. PCR was conducted using AmpliTaq Gold™ 360 (Thermo) according to the manufacturer’s instructions. Primers for the TLR3 mutation will be provided upon request. The PCR products were purified using the ExoSAP protocol, sequenced directly with the Big Dye Terminator Cycle Sequencing Kit v3.1 (Thermo Fisher Scientific, Waltham, MA), and analyzed by capillary electrophoresis using the 3500 Genetic Analyzer.

### Immunofluorescence analysis

hiPSCs were fixed in 100% methanol at −20 °C for 7 min or 4% paraformaldehyde for 20 min at RT and incubated with primary antibodies OCT4 (1:25 Novus Biological, Centennial, CO, USA), SOX2 and NANOG. Successively incubated with specific Alexa Fluor 568 and 488-labeled secondary antibodies (Invitrogen, Carlsbad, CA, USA) in the presence of Hoechst 33342 (Sigma-Aldrich). Slices were analyzed under fluorescence microscopy, and images were acquired using a Zeiss (Zeiss, Thornwood, NY USA) Axioplan 2 microscope and Leica TCS SP5 confocal microscope (Leica, Wetzlar, Germany).

### STR analysis

Short tandem repeat (STR) analysis of 24 *loci* was performed using the GlobalFiler™ PCR Amplification Kit (Life Technologies), according to the manufacturer’s instructions. The separation and detection of the amplicons were conducted by Applied Biosystems® 3130xl Genetic Analyzer (Thermo Fisher Scientific, Waltham, MA, USA) according to the manufacturer’s protocols. The collection and the analysis of data by CE were assessed by GeneMapper™ ID-X Software v1.5 (Applied Biosystems).

### Karyotyping

hiPSCs were treated with 10 µg/ml colcemid (BioWest USA, Inc., Bradenton, FL, USA) for 3 h at 37 °C before being harvested using trypsin-EDTA 1X solution (Euroclone S.p.A., Italy). They were resuspended in a hypotonic solution 0.075 M KCl (Sigma-Aldrich™, USA) and incubated at 37 °C for 10 min, then fixed in 3:1 methanol: glacial acetic acid (both from Sigma Aldrich™, USA) at room temperature for 10 min. hiPSCs were then centrifuged and fixed three times (10 min at room temperature, 10 min at room temperature and overnight at 4 °C). Slides were allowed to age for 24 h (60 °C) before the staining with Giemsa (Sigma Aldrich™, USA) and G banding, according to the protocol by Seabright et al. with modifications. Chromosome analysis was carried out on 20 metaphases. The karyotype was expressed following the guidelines of the International System for Human Cytogenomic Nomenclature (2020) (ISCN2020) using software dedicated to karyotyping: Ikaros Karyotyping Software, version 6.3.9 (MetaSystems, Germany).

### Mycoplasma test

Mycoplasma contamination was assessed with the MycoSPY® Master Mix (M020-025; Biontex, Munchen, Germany) following the manufacturer’s instructions.

### Telomere length analysis

Telomere length was measured using the protocol described by De Benedittis et al. [23]. Ct values were determined in each sample during the qPCR run. Relative telomere length was estimated by comparing the amount of telomere repeat amplification product (T, TEL) to a single copy gene (S, β-globin) product. The T/S ratio provided a relative measure of telomere length relative to the reference gene, facilitating comparisons between samples and experimental conditions.

### Differentiation into Human 3D lung organoids (hLORGs)

Lung progenitor cells were generated using the STEMdiff Lung Progenitor Kit (Stemcell Technologies, cat. # 100-0230, Vancouver, Canada), a serum-free medium system optimized for the efficient and reproducible generation of lung progenitor cells, as reported in Spitalieri et al. Resulting 3D alveolospheres were matured and maintained in culture by successive passages, re-embedding every two weeks in Matrigel GFR (Corning, New York, NY, USA). 60-day-old lung organoids were used to characterize specific markers expression and SARS-CoV-2 infection studies.

### hLORG Characterization

Immunofluorescence was performed on hLORG embedded in OCT [8]. After permeabilization (10 minutes in 0.2% Triton X-100), samples were blocked for 30 min in 1% BSA and incubated with primary antibodies for 1 h at room temperature (mouse anti-tubulin 1: 1000 and rabbit anti-actin 1:200, Merk, Darmstadt, Germany; mouse anti-DPP4 1:50, BioLegend, San Diego, California, CA, USA). The sections were analyzed under a fluorescence microscope (Eclipse E 600; Nikon, Tokyo, Japan), and images were acquired using a digital camera and ACT-1 software (Nikon, Tokyo, Japan).

Immunohistochemistry was performed on the organoids fixed in 4% formalin (Spitalieri et al.), using goat anti-ACE2 antibody (1:60, AF 933, R & D Systems, Minneapolis, Minnesota, MN, USA) and rabbit anti-SFTPC (1:1000, ab 3786, Chemicon, Temecula, California, CA, USA). Images were acquired with the Eclipse E600 microscope.

### Pseudotyped SARS-CoV-2-S Infection

hLORGs were exposed to 50 μL of VSVpp.SARS-CoV-2-S virus (ReVacc) and incubated at 37 °C with 5% CO_2_ for 2 h, followed by continued incubation in Matrigel GFR for a total of 48 hours before analysis [8].

The infections with the Pseudotyped SARS-CoV-2-S were assessed at 48 h post-infection for luciferase activity alone, following the manufacturer’s instructions. The activity of firefly luciferase was quantitatively measured using the Promega Luciferase Assay System (Promega, Madison, Wisconsin, WI, USA, E1501), and luminescence was evaluated with a luminometer, following the manufacturer’s instructions.

### SARS-CoV-2 isolation and production

SARS-CoV-2 (hCoV-19/Italy/CDG1/2020/EPI_ISL_412,973) was isolated from nasopharyngeal swab by the Department of Infectious Diseases, National Institute of Health Rome, Italy. The virus was then propagated in African green monkey kidney (Vero-E6) cells cultured in MEM containing 2% FBS. 72 h after the infection, supernatants containing the released viral particles were collected and centrifuged at 600 *g* for 5 min. The viral titer was evaluated by plaque assay. Virus stocks were stored at −80 ^◦^C until use.

All the experiments with SARS-CoV-2 were performed in the BSL3 lab at the Defense Institute for Biomedical Sciences (ISBD).

### SARS-CoV-2 infection

hLORGs were detached from the plate and treated with trypsin for one minute, in order to open the alveolospheres and to facilitate the entry of the virus into the ones. Then, alveolospheres were inoculated with SARS-CoV-2 at multiplicity of infection (m.o.i.) of 0.01 and incubated for 1 h at 37 °C with gentle shake after 30 min. Mock infection was performed with culture medium only. After the viral challenge, viral inoculum was removed and the alveolospheres were plated in Matrigel GFR-containing medium for 48 and 72 h. Subsequently, supernatants and hLORGs were collected and subjected to different analyses.

### Plaque assay

Viral titer was quantified by plaque assay. Confluent monolayers of Vero-E6 cells were inoculated with 10-fold dilutions of the supernatant from infected hLORGs and incubated for 1 h at 37 °C. Mock infection was performed with MEM medium. After viral adsorption period, the inoculum was removed. The cell monolayer was overlaid with a mixture of MEM (no glutamine, no phenol-red-GIBCO), 1.5% Tragacanth (SIGMA), NaHCO3 7% (Gibco), L-glutamine 1X (Gibco), MEM NEAA 1x (Gibco), 0.02 M Hepes (Euroclone), DMSO (Sigma-Aldrich) and 2% FBS (final concentration). Five days after infection, the mixture was carefully removed, wells were washed with saline solution, and stained with 1% crystal violet for 20 min before counting plaque-forming units (PFU). The virus titer was calculated as PFU/ml.

### TLR pathway gene expression analysis

One µg of RNA was reverse transcribed, and SYBR Green Master Mix was used to assay *TLR* mRNAs (Life Technologies Corporation, Foster City, CA, USA). GAPDH was employed as a reference gene. Primer sequences are available upon request. The 2 − (ΔCt) and comparative ΔΔCt methods were used to quantify relative *TLR* pathway gene expression levels.

### TLR3 protein expression

At 48 h post infection, hLORGs were detached, washed with culture medium and centrifuged at 300 rpm for 5 min. The pellet was lysed in cold lysis buffer containing protease and phosphatase inhibitors. Lysates were centrifuged at 13,000 *g* for 30 min at 4 °C to remove debris, diluted in SDS sample buffer containing dithiothreitol (DTT) and placed on a thermoblock at 100 °C for 5 min. Cell pellets were processed in the BSL-3 laboratory before being separated by SDS-PAGE and transferred to nitrocellulose membranes.

Western blot analysis for TLR3 was performed as described in Sax et al. Briefly, 50 µg of total protein was separated by 4–12%SDS-PAGE (Thermofisher, Waltham, MA, USA), electroblotted and incubated with specific primary antibodies overnight at +4 °C: rabbit α-TLR3 (D10F10; dil 1:1000; Cell Signaling, Danvers, MA, USA). Densitometric analysis was performed for two independent experiments using ImageJ software (bundled with 64-bit Java 8; https://imagej.net/ij/index.html), and monoclonal mouse α-Vinculin (S-V9131; 1:1000; Sigma Aldrich, St. Louis, Missouri, USA) was used as the normalizing antibody. See Blue Plus 2 Pre-Stained Protein Ladder (3 to 198 kDa, Thermo Fisher Scientific) was used for all SDS-PAGE.

### RNA-Seq analysis

Total RNA was quantified using the Qubit 4.0 fluorimetric Assay (Thermo Fisher Scientific). Libraries were prepared from 250 ng of total RNA using the NEGEDIA Digital mRNA-seq clinical grade sequencing service v2.0 (NEGEDIA srl.), which included library preparation, quality assessment and sequencing on a NovaSeq 6000 sequencing system using a single-end, 100-cycle strategy (Illumina Inc.).

The raw data were analysed by NEGEDIA srl. proprietary NEGEDIA Digital mRNA-seq pipeline (v2.0), which involves a cleaning step by quality filtering and trimming, alignment to the reference genome and counting by gene. The raw expression data were normalized, analysed by the NEGEDIA DEG analysis pipeline (v1.2.0) and visualized in a proprietary report (v1.0).

### Statistical analyses

Molecular experiments and luciferase assay were performed in technical triplicate, and data were analyzed using GraphPad Prism version 10 and SPSS version 27 (IBM Corp, USA). Paired Student’s *t* test and a one-way ANOVA were used to assess group differences. Values shown in figures represent the means of three independent experiments ±standard deviation (SD). Statistical significance was established at * *p* < 0.05, ** *p* < 0.01, and *** *p* < 0.001.

## Supplementary information

Supplementary Material

Supplementary Material

Supplementary Material

Uncropped gel

## Data Availability

The authors declare that RNA sequencing data are not publicly available due to patient privacy, but all other data supporting the findings of this study are available within the article and its supplementary material files, or from the corresponding author upon reasonable request.
