# Supplementary figures and images for: Modelling severe COVID-19 in TLR3-mutated hiPSCs-derived lung organoids
Source: Cell Death Discov. 2025 Dec 26;12:74. doi: 10.1038/s41420-025-02936-5 (PMC12858893; doi:10.1038/s41420-025-02936-5)

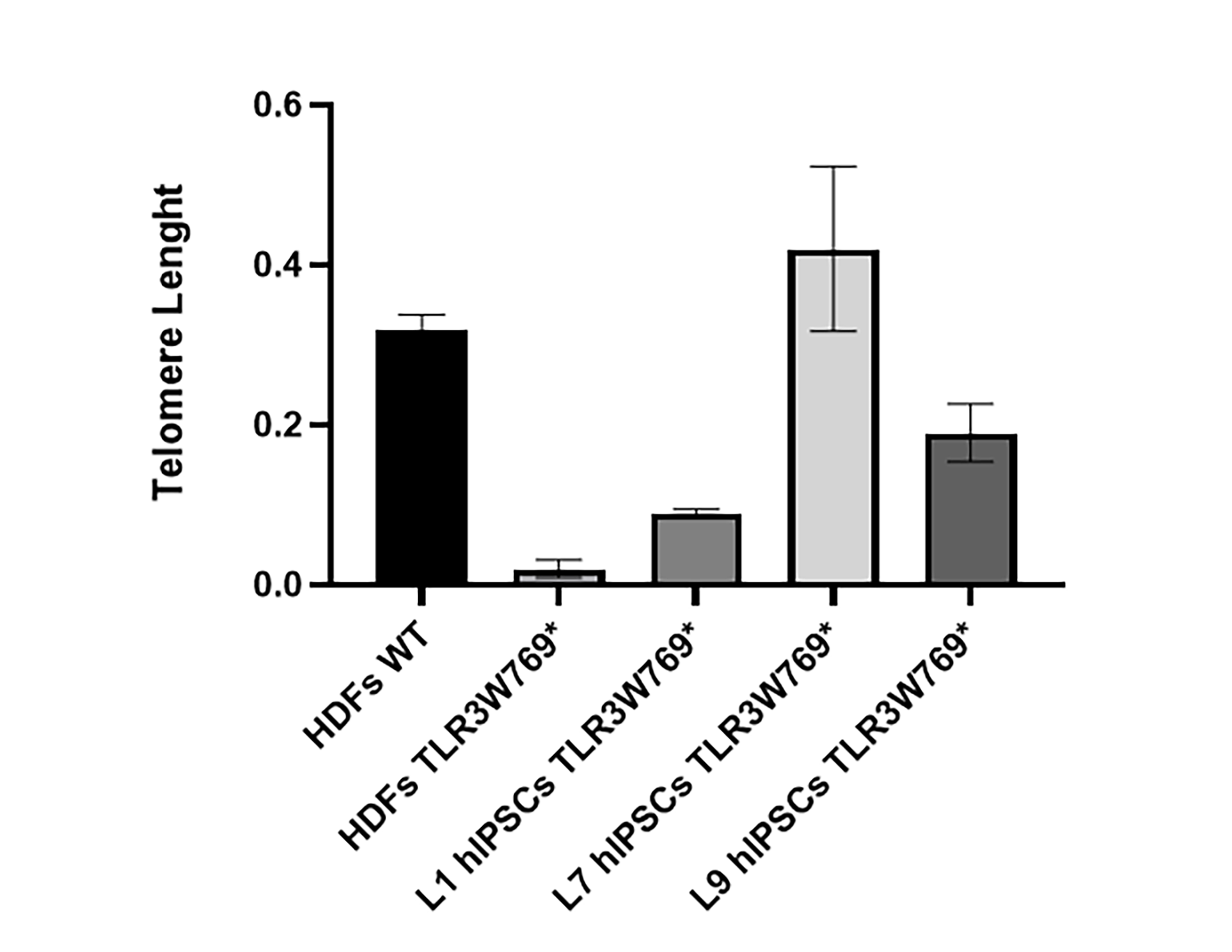

Supplement: Supplementary file 1 — Supplementary Material [file 41420_2025_2936_MOESM1_ESM.tif]

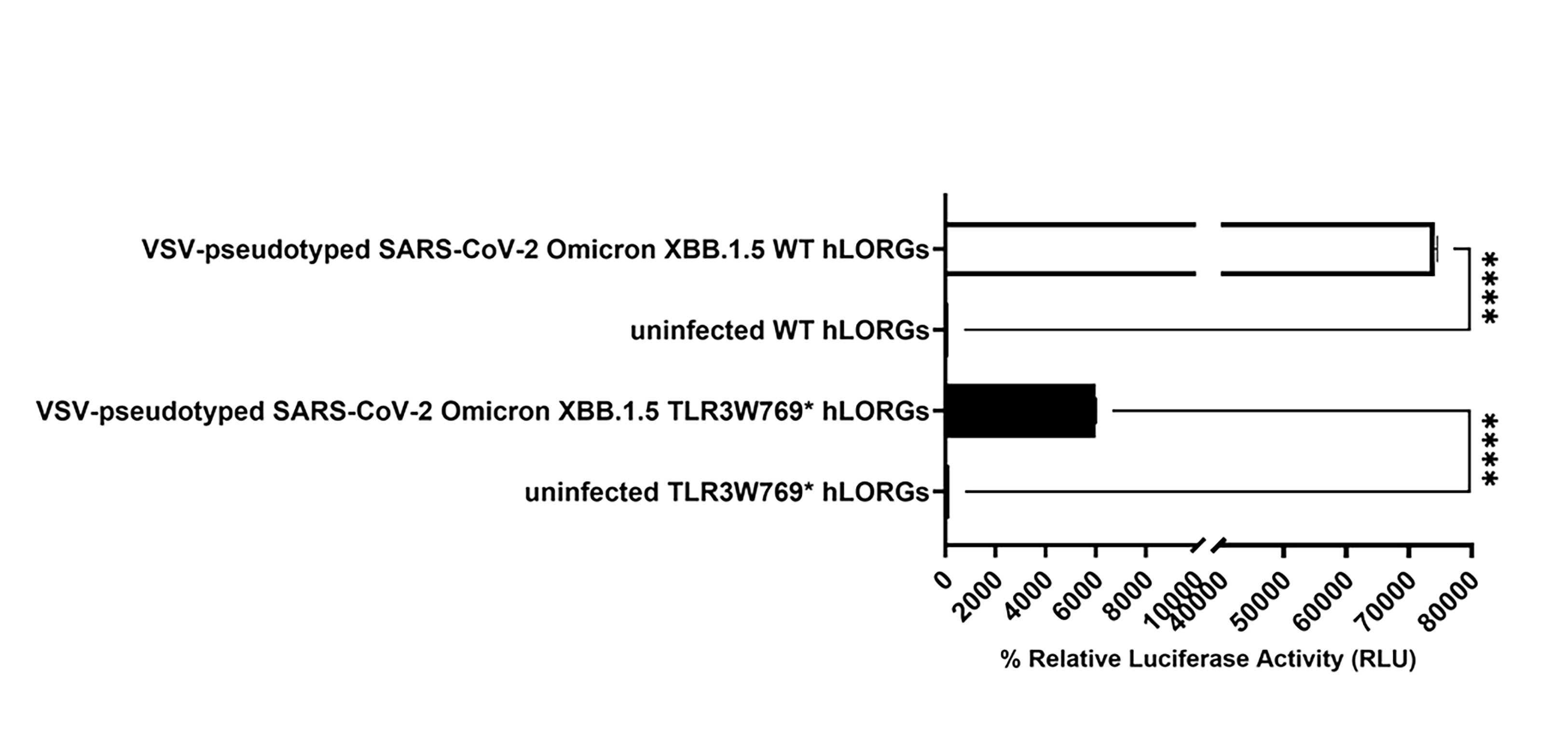

Supplement: Supplementary file 2 — Supplementary Material [file 41420_2025_2936_MOESM2_ESM.tif]

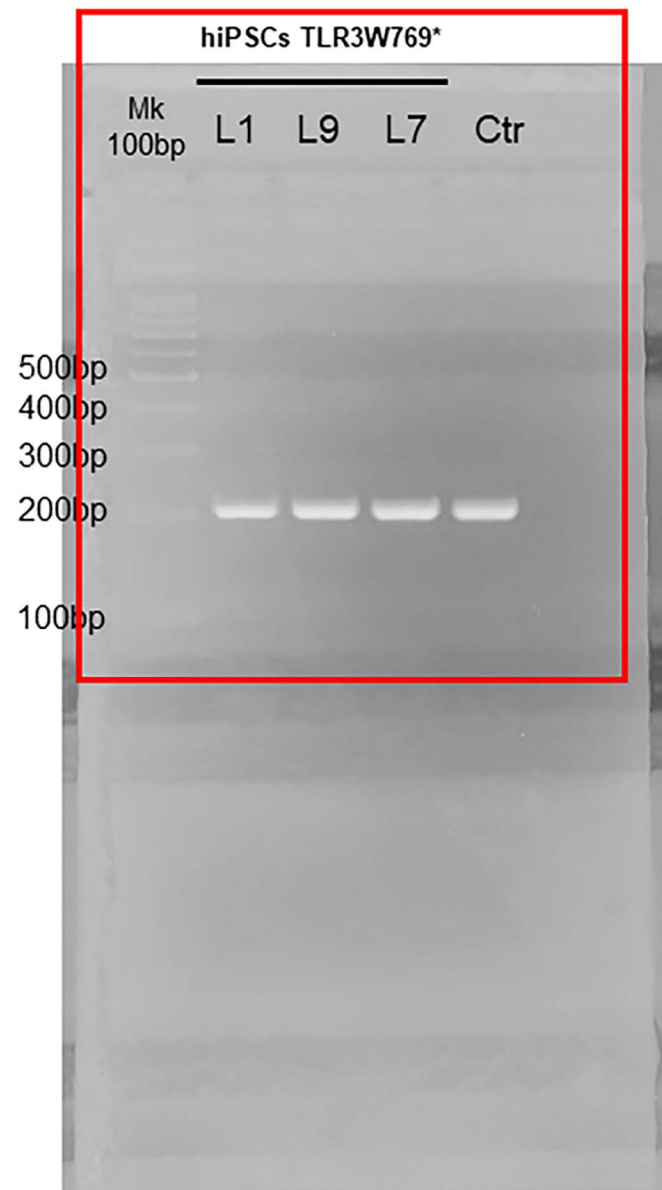

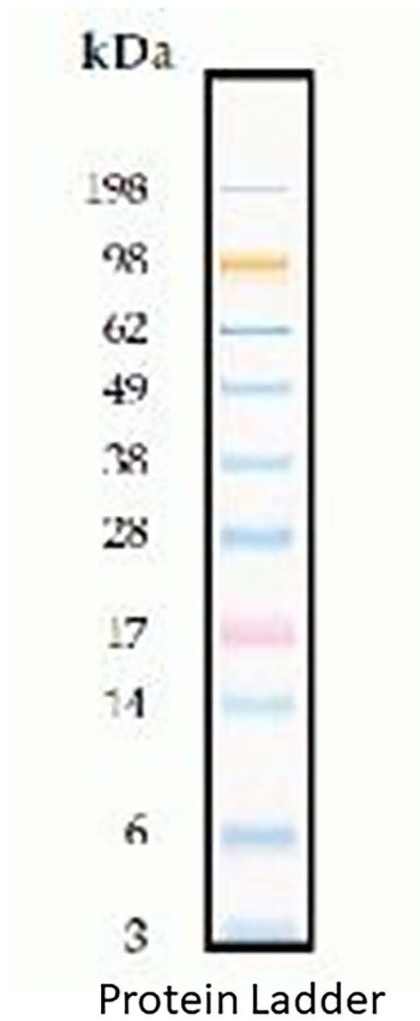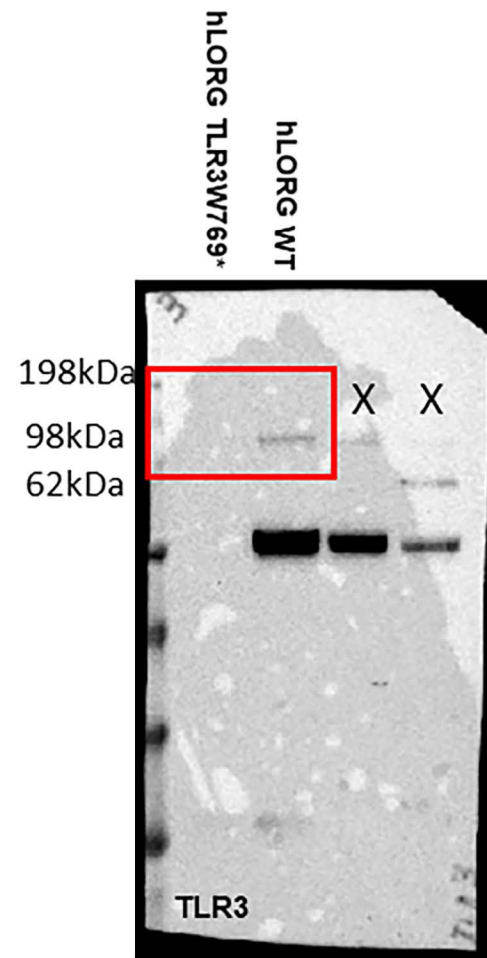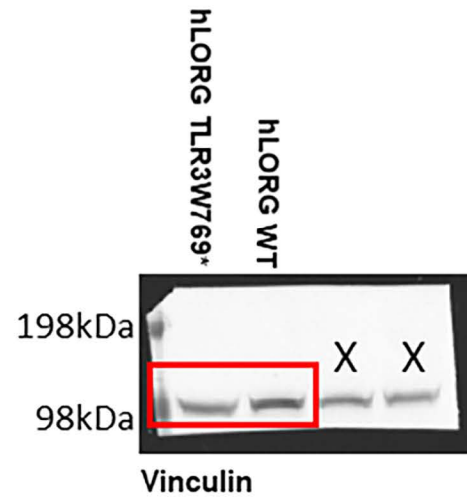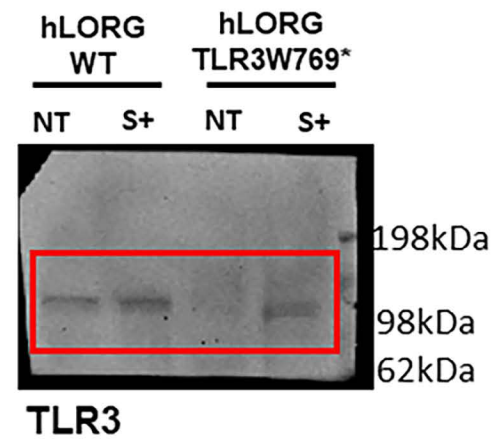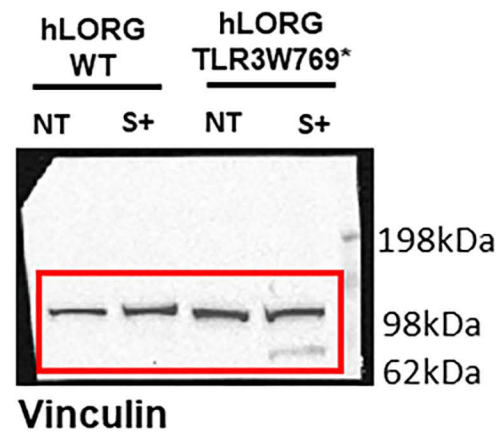

Supplement: Supplementary file 4 — Uncropped gel [file 41420_2025_2936_MOESM4_ESM.pdf]
