# Supplementary material for: Modelling severe COVID-19 in TLR3-mutated hiPSCs-derived lung organoids
Source: Cell Death Discov. 2025 Dec 26;12:74. doi: 10.1038/s41420-025-02936-5 (PMC12858893; doi:10.1038/s41420-025-02936-5)
Supplement: Supplementary file 3 — Supplementary Material [file 41420_2025_2936_MOESM3_ESM.docx]

**Supplementary Material**

***Table* S1 Genetic profiling of the generated hiPSCs from TLR3W769* patients through the STR analysis showed that the hiPSCs from each individual are typically identical, referred to as the same donor HDFs TLR3W769***

| Locus designation | Chromosome location | HDFs  TLR3W769* | hiPSCs TLR3W769*  L1 | hiPSCs TLR3W769*  L7 | hiPSCs TLR3W769*  L9 |
| --- | --- | --- | --- | --- | --- |
| D3S1358 | 3p21.31 | 14;17 | 14;17 | 14;17 | 14;17 |
| vWA | 12p13.31 | 14;18 | 14;18 | 14;18 | 14;18 |
| D16S539 | 16q24.1 | 10;11 | 10;11 | 10;11 | 10;11 |
| CSF1PO | 5q33.3-34 | 10;11 | 10;11 | 10;11 | 10;11 |
| TPOX | 2p23-2per | 8;11 | 8;11 | 8;11 | 8;11 |
| Y indel | Yq11.221 | 2 | 2 | 2 | 2 |
| Amelogenin | X: p22.1-22.3  Y: p11.2 | X;Y | X;Y | X;Y | X;Y |
| D8S1179 | 8q24.13 | 13;14 | 13;14 | 13;14 | 13;14 |
| D21S11 | 21q11.2-q21 | 29;30 | 29;30 | 29;30 | 29;30 |
| D18S51 | 18q21.33 | 10;20 | 10;20 | 10;20 | 10;20 |
| DYS391 | Yq11.21 | 10 | 10 | 10 | 10 |
| D2S441 | 2p14 | 11;14 | 11;14 | 11;14 | 11;14 |
| D19S433 | 19q12 | 14;15 | 14;15 | 14;15 | 14;15 |
| TH01 | 11p15.5 | 7;9 | 7;9 | 7;9 | 7;9 |
| FGA | 4q28 | 22;22 | 22;22 | 22;22 | 22;22 |
| D22S1045 | 22q12.3 | 11;15 | 11;15 | 11;15 | 11;15 |
| D5S818 | 5q21-31 | 11;12 | 11;12 | 11;12 | 11;12 |
| D13S317 | 13q22-31 | 12;13 | 12;13 | 12;13 | 12;13 |
| D7S820 | 7q11.21-22 | 8;12 | 8;12 | 8;12 | 8;12 |
| SE33 | 6q14 | 17;17 | 17;17 | 17;17 | 17;17 |
| D10S1248 | 10q26.3 | 14;14 | 14;14 | 14;14 | 14;14 |
| D1S1656 | 1q42.2 | 16;16 | 16;16 | 16;16 | 16;16 |
| D12S391 | 12p13.2 | 19;20 | 19;20 | 19;20 | 19;20 |
| D2S1338 | 2q35 | 17;19 | 17;19 | 17;19 | 17;19 |

|  | |  |  |  |
| --- | --- | --- | --- | --- |
| **Table S2 a. Up-regulated genes in WT versus TLR3W769* hLORGs after infection** | | | | |
| **Gene id** | **Gene name** | **Pvalue** | **Padj** | **Log2FoldChange** |
| ENSG00000232533 | *AC093673.5* | 0.00000263034 | 0.001829886 | 7.246.021 |
| ENSG00000162772 | *ATF3* | 7,0397E-09 | 4,65254E-06 | 2.047.614 |
| ENSG00000113734 | *BNIP1* | 0.0001435526 | 0.02342566 | 1.884.676 |
| ENSG00000275302 | *CCL4* | 0.0003474644 | 0.04188258 | 212.051 |
| ENSG00000172361 | *CFAP53* | 0.0001816906 | 0.02825396 | 2.999.873 |
| ENSG00000104324 | *CPQ* | 0.00007864901 | 0.0169471 | 4.242.424 |
| ENSG00000261188 | *CTA-445C9.14* | 0.00004591565 | 0.01187524 | 5.046.581 |
| ENSG00000078401 | *EDN1* | 1,94445E-07 | 0,006425434 | 3.027.287 |
| ENSG00000153266 | *FEZF2* | 0.000004467804 | 0.002582567 | 7.478.291 |
| ENSG00000122641 | *INHBA* | 0.0004345314 | 0.0497003 | 1.878.546 |
| ENSG00000100097 | *LGALS1* | 0.000001240146 | 0.001024516 | 5.210.831 |
| ENSG00000139625 | *MAP3K12* | 0.0003129316 | 0.03986554 | 1.864.734 |
| ENSG00000008130 | *NADK* | 0.000137125 | 0.02317096 | 190.402 |
| ENSG00000053438 | *NNAT* | 0.0003433113 | 0.04188258 | 2.147.805 |
| ENSG00000179299 | *NSUN7* | 0.0002338819 | 0.03288778 | 2.589.859 |
| ENSG00000274979 | *RP11-1143G9.5* | 0.0003252293 | 0.04064401 | 2.068.357 |
| ENSG00000129566 | *TEP1* | 0.000003426647 | 0.00215683 | 7.169.568 |
| ENSG00000167920 | *TMEM99* | 0.0001292344 | 0.02277626 | 3.174.931 |
| **Table S2 b. Down-regulated genes in WT versus TLR3W769* hLORGs after infection** | | | | |
| **Gene id** | **Gene name** | **Pvalue** | **Padj** | **Log2FoldChange** |
| ENSG00000154265 | *ABCA5* | 0.000001133347 | 0.0009987056 | -2.750.313 |
| ENSG00000173467 | *AGR3* | 0.0002083349 | 0.03026122 | -2.721.224 |
| ENSG00000214274 | *ANG* | 0,3748668 | 0.0004129157 | -7.417.496 |
| ENSG00000213213 | *CCDC183* | 0.0002063863 | 0.03026122 | -2.761.846 |
| ENSG00000239704 | *CDRT4* | 0.0002322756 | 0.03288778 | -2.571.701 |
| ENSG00000111817 | *DSE* | 0.000005112537 | 0.002599135 | -6.917.771 |
| ENSG00000112319 | *EYA4* | 0.00004090173 | 0.01132201 | -5.051.657 |
| ENSG00000171560 | *FGA* | 0,1191864 | 0.0001575405 | -721.437 |
| ENSG00000171557 | *FGG* | 0,02929033 | 0.00004839495 | -2.039.488 |
| ENSG00000179344 | *HLA-DQB1* | 0.00002714737 | 0.008889338 | -5.250.171 |
| ENSG00000128422 | *KRT17* | 0.00001654616 | 0.006075198 | -3.564.637 |
| ENSG00000112818 | *MEP1A* | 0.0001382041 | 0.02317096 | -3.415.092 |
| ENSG00000160588 | *MPZL3* | 0.0003259392 | 0.04064401 | -2.217.772 |
| ENSG00000280623 | *PCAT14* | 0.000003252534 | 0.0021496 | -6.522.358 |
| ENSG00000154553 | *PDLIM3* | 0.0000291728 | 0.009057733 | -406.544 |
| ENSG00000138308 | *PLA2G12B* | 0.0003485462 | 0.04188258 | -2.111.152 |
| ENSG00000008323 | *PLEKHG6* | 0.00004339446 | 0.01147176 | -5.106.192 |
| ENSG00000115718 | *PROC* | 0.0003400655 | 0.04188258 | -2.128.186 |
| ENSG00000149177 | *PTPRJ* | 0.0002605062 | 0.03520038 | -2.478.248 |
| ENSG00000225177 | *RP11-390P2.4* | 0.00005478819 | 0.01293197 | -4.788.879 |
| ENSG00000276136 | *RP11-50I19.2* | 0.0003136644 | 0.03986554 | -2.265.509 |
| ENSG00000163993 | *S100P* | 4,87921E-10 | 6,44934E-06 | -3.190.541 |
| ENSG00000129158 | *SERGEF* | 0.00024358 | 0.03353792 | -2.590.897 |
| ENSG00000100665 | *SERPINA4* | 0.00007298952 | 0.01635213 | -4.358.891 |
| ENSG00000132386 | *SERPINF1* | 0.0001187264 | 0.02210317 | -3.406.732 |
| ENSG00000146039 | *SLC17A4* | 0.0001213647 | 0.02228054 | -2.725.695 |
| ENSG00000122696 | *SLC25A51* | 0.0001272822 | 0.02277626 | -355.105 |
| ENSG00000227533 | *SLC2A1-AS1* | 0.0001424248 | 0.02342566 | -337.853 |
| ENSG00000133710 | *SPINK5* | 0,08242613 | 0.0001210565 | -2.422.335 |
| ENSG00000135111 | *TBX3* | 0,002938763 | 0.000005549225 | -1.767.249 |
| ENSG00000088992 | *TESC* | 0.00001399438 | 0.005285076 | -3.969.747 |
| ENSG00000169903 | *TM4SF4* | 0.00001949535 | 0.006607425 | -5.161.968 |
| ENSG00000168016 | *TRANK1* | 0.0001785973 | 0.02810356 | -3.040.884 |
| ENSG00000178764 | *ZHX2* | 0.000009451389 | 0.004100325 | -6.569.839 |
| ENSG00000167562 | *ZNF701* | 0.00001355674 | 0.005270383 | -6.355.618 |
| ENSG00000160336 | *ZNF761* | 0.00000562978 | 0.00275609 | -6.886.751 |


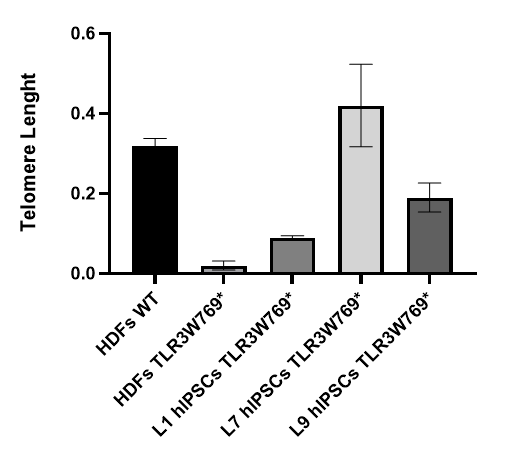


***Figure S1. Telometer length in hiPSCs TLR3W769*.*** Relative telomere length was evaluated by real-time q-PCR in three lines (L1, L7 and L9) of hiPSCs TLR3W769* compared to the same fibroblast donor HDFs TLR3W769*. Values were normalised to telomere expression relative to fibroblasts from a 46-year-old patient (HDFs WT). Experiments were carried out in triplicate. Error bars indicate the standard deviation ± standard error of the mean (SEM)


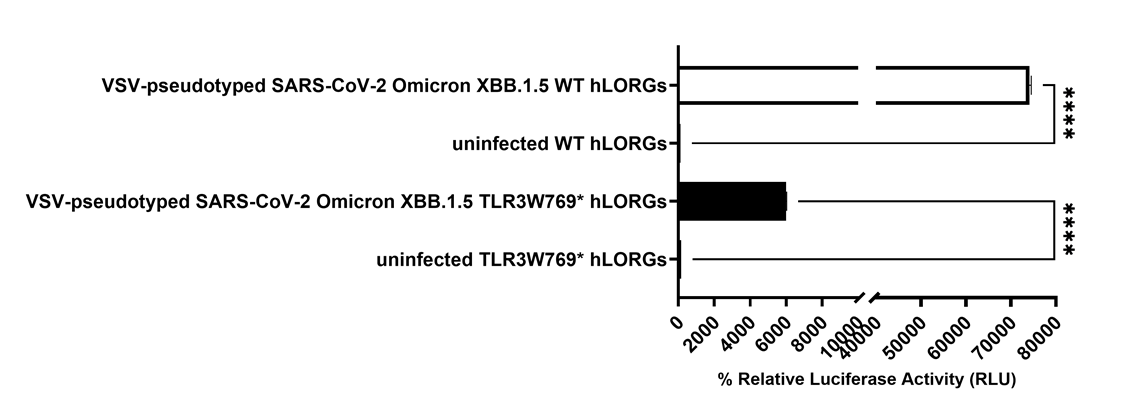


***Figure S2:*** Transduction efficiency was quantified by measuring virus-encoded luciferase activity in in TLR3W769* hLORG and WT hLORG infected for 48 h with pseudotypes VSV-pseudotyped SARS-CoV-2 Omicron XBB.1.5 . Data are expressed as the percentage of infection, and the average data from two biological replicates are presented. Error bars indicate the standard deviation (±SD). **** p < 0.0001 by one-way ANOVA test
